# Patterns of knowledge and condom use among population groups: results from the 2005 Ethiopian behavioral surveillance surveys on HIV

**DOI:** 10.1186/1471-2458-8-429

**Published:** 2008-12-31

**Authors:** Getnet M Kassie, Damen H Mariam, Amy O Tsui

**Affiliations:** 1 Addis Ababa University, School of Public Health, Addis Ababa, Ethiopia; 2 Johns Hopkins University, School of Public Health, Department of Population and Family Health Sciences, Baltimore, MD, USA

## Abstract

**Background:**

Behavioral surveys help interpret the magnitude of HIV/AIDS. We analyzed indicators of knowledge on HIV/AIDS and condom use among sub populations selected for behavioral surveillance in Ethiopia.

**Methods:**

We used 2005 HIV/AIDS behavioral data from ten target groups. These were female sex workers, defense forces, police force, pastoralists, truck drivers, intercity bus drivers, road construction workers, teachers, factory workers and people in ANC catchment areas.

**Results:**

Data from 14,524 individuals were analyzed. The majority were males (63.6%). Overall, knowledge of the three preventive methods, misconceptions and comprehensive knowledge was 57%, 75% and 18.5%, respectively. Female sex workers and the defense force showed some behavioral change in using a condom during the most recent sexual encounter and consistently used a condom with non-regular sexual partners and paying partners. Women, pastoralists and the illiterate were less likely to use condom.

**Conclusion:**

Misconceptions about the transmission of HIV were high and comprehensive knowledge about HIV & AIDS was low, particularly among pastoralists. Consistent condom use and condom use during the last sexual encounter were high among both female sex workers and defense force employees, both with paying and non-regular sexual partners. This might be a positive sign, though a considerable proportion in each target group did not report using a condom during sex with non-regular partners.

## Background

More than two decades have passed since the evidence of HIV in Ethiopia [1,2]. In spite of the indications that the HIV prevalence is leveling off, Ethiopia has remained one of the countries with a high number of people living with HIV/AIDS [3]. Because of the significant variation of prevalence estimates of HIV between the 2005 Ethiopian Demographic and Health Survey (1.4%) [4] and the 2005 ANC sentinel surveillance report (3.5%) [5], the Ministry of Health had to synthesize the findings in both and agree on a single point national estimate of the HIV adult prevalence which was 2.1% with 7.7%, and 0.9% urban and rural prevalence, respectively [6]. One of the major challenges in this regard was lack of serial quality data on high-risk and sexual behaviors as related to HIV/AIDS and sexually transmitted infections (STIs) that would contribute to the interpretation of the patterns of magnitude of HIV/AIDS [7].

Among the earliest groups that were identified and followed for HIV surveillance were female sex workers, long distance truck drivers, uniformed services and mobile groups [8-10]. In addition to the high risk-groups mentioned above, youth, teachers, road construction workers, the pastoralist community along transport corridors, people in Ante Natal Care (ANC) catchment areas, and other mobile populations such as intercity bus drivers were added to the list of potential risk groups for behavioral HIV surveillance. The selection of the target groups was based on emerging data and recommendations by the national technical working group for HIV surveillance.

Conventional household surveys usually fall short of penetrating high-risk groups which often shape the HIV epidemic. Behavioral Surveillance Surveys (BSS) employ sampling methods that better capture hard-to-reach populations through the use of standardized methodologies and tools that help comparison and monitoring of risk behaviors over time [11,12]. Ethiopia has already conducted two rounds of behavioral surveys, one in 2002 and another in 2005. Both rounds of surveys revealed a high level of awareness about HIV/AIDS [13,14]; however, the level of comprehensive knowledge was very low. For example, the level of comprehensive knowledge among long truck drivers and intercity bus drivers where the same methodology was used was lower in 2005 than in 2002 (42.8% vs. 29% and 31.1% vs. 23.2% respectively). Comprehensive knowledge was measured by six questions that indicate *knowledge of the three preventive methods *and *absence of misconceptions*. Out of the three common misconceptions one was universally recommended while two of them were specific to Ethiopia.

Changes in behaviors of people measured by behavioral surveys may indicate the effects of the several interventions aimed at reducing high-risk behaviors. One of the important assumptions in studying knowledge and misconceptions is people who are informed and equipped with necessary skills make better decisions in protecting themselves against HIV infection. Hence, along with other data sources, information garnered from behavioral surveillance surveys can illustrate trends in risk behaviors among various segments of the population and thereby better inform program interventions [12].

The objective of this analysis is to measure the level of essential knowledge on HIV/AIDS and condom utilization among several Ethiopian subpopulations selected for behavioral surveillance. By analyzing the pattern of behaviors and knowledge among these subgroups, practitioners and decision makers will be able to better calibrate interventions to reach these populations.

## Methods

This study used behavioral data on HIV/AIDS/STIs conducted in 2005 in Ethiopia. The data was obtained from cross-sectional surveys on ten target populations for HIV/AIDS/STIs behavioral surveillance surveys.

BSS are repeated cross-sectional surveys that use probability sampling mehods on selected population groups that are hard-to-reach by conventional househod surveys. The BSS guideline provides a standardized methodology and generic questionnaires that are adapted to local situations [11]. For example, mobile populations (Long distance drivers) are sampled using time-location sampling through extensive mapping and listing subject locations, as well as peak and low congregation times of the target group. On the other hand, for sampling people in ANC catchement areas a houdehold survey was used. This was done after the ANC catchement areas were categorised as high, medium and low prevelence sites for HIV based on ANC surveillance reports.

### Target populations and sites

Selection of target groups was based on the distribution of HIV in different subpopulations and the relevance of the groups in spreading and maintaining HIV/AIDS sexual networks based upon location, mobility or priorities of interventions. Table 1 shows the target populations, number of sites, size of the sample and sampling methods.

**Table 1:** Distribution, sample size and sampling method for study population, Ethiopia 2005

Target population	Data (Sample interviewed)	Sampling method	Number of sites and primary sampling unit
Long Distance Truck drivers	600	Time-location clusters	5 truck weighting sites

Inter City Bus Drivers	600	Time-location clusters	1 central site

Female Sex Workers	1398	Time-location clusters	3 urban centers

Pastoralists	1608	Two – stage sampling Respondents selected using systematic random sampling	2 regions- villages

Factory Workers	416	Systematic random sampling	1 factory

Defense forces	1305	Two – stage sampling Respondents selected using systematic random sampling	2 sites – brigade

Population in ANC catchment areas*	5207	Two – stage sampling Respondents selected using systematic random sampling	7 sites – enumeration area

Road CW	1623	Two – stage sampling Respondents selected using systematic random sampling	7 sites, 4 directions – construction sites

Police force	1071	Two – stage sampling Respondents selected using systematic random sampling	7 sites – police stations

Teachers	696	Two – stage sampling Respondents selected using systematic random sampling	11 regions – schools

Total	14524		

* = 7 sites were selected out of 82 ANC sentinnel sites (8.5%)

### Sample sizes, sampling and survey procedures

#### Sample size

In calculating the sample size for each target group assumptions and findings of the first round BSS were used. Sample size determination and sampling procedures followed guidelines for BSS. Design effect and power were considered in estimating the sample size for each corresponding target group.

### Instruments and Data Collection procedures

#### Instrument

The BSS uses standardized questionnaires that are pre-tested and adapted to the Ethiopian situation. Almost all the questions are the same for all targets except a few that are specific to each target group. All the questions were closed ended with options provided. The questionnaires were translated into major languages namely *Amharic, Oromifa*, and *Afarigna *and back translated to check for consistency with the English version. The questionnaires were field tested before use.

The data collectors had completed high school and most were from the Central Statistical Agency (CSA). In order to ensure confidence and convenience, the interviewers were sex matched with interviewees and were selected from different areas of the same region speaking the same language as the interviewees. This arrangement was preferred to avoid possibilities of acquaintance between the interviewer and interviewee.

#### Variables

*a) Explanatory variables: *Age, sex, education and target group *b) the outcome variables included: *knowledge of prevention methods, misconception, comprehensive knowledge, consistent condom use with non regular sexual partner and paying sexual partner; condom use during last sexual encounter with non regular sexual partner and paying sexual partner.

#### Data Quality

The BSS used highly supervised data collection and data handling procedures that followed manuals of the CSA. One supervisor supervised a maximum of four to six data collectors and each questionnaire was checked for completeness and consistency on the spot. All data collected were handled and processed by CSA.

#### Data analysis

Data was analyzed using two statistical softwares: SPSS version 13 and Stata version 9. The analysis focused on assessing knowledge and condom use variables as related to the different target groups. We used bivarite analysis and then logistic regression controlling for age, sex and education levels. Adjusted Odds Ratios and 95% Confidence Intervals were calculated to indicate statistical associations at 5% significance level.

The study was approved by the Research and Publication Committee of the Faculty of Medicine, Addis Ababa University and the BSS by the Ethiopian Science and Technology Agency.

### Operational definitions

#### Knowledge of prevention methods

Knowledge of prevention included three questions measuring Abstinence, Be faithful and Condom use:

1. Can people protect themselves from HIV, the virus that causes AIDS by using condom correctly every time they have sex?

2. Can people protect themselves from HIV by having one uninfected faithful sexual partner? (Excluding other transmission routes).

3. Can people protect themselves from HIV by abstaining from sexual intercourse? (Excluding other transmission routes).

A person was classified as knowledgeable on prevention if s/he mentioned all three methods.

#### Misconceptions to three

Knowledge on misconceptions was measured if a person rejected three common misconceptions. The first two of the misconceptions are specific to Ethiopia:

1. Can a person get the HIV virus from eating uncooked eggs from a chicken that swallowed a condom?

2. Can a person get the HIV virus from eating raw meat prepared by a person infected by HIV?

3. Do you think that a healthy-looking person can be infected with HIV, the virus that causes AIDS?

#### Comprehensive knowledge

this index was built based on the answers to six questions i.e. three questions on knowledge of prevention and three questions on common misconceptions. A person was considered as having had comprehensive knowledge if s/he knew all the three preventive methods and rejected the three misconceptions.

#### People in the ANC catchment area

Refers to the general population living in areas where ANC surveillance is taking place in the country. It included both rural and urban populations living in ANC surveillance sites.

#### Paying sexual partner

Having sex with someone in exchange for money.

#### Non-regular sexual partner

Non-commercial sexual partnership for unmarried or non-cohabitating partners.

#### Defense forces

Includes ground forces and air force

#### Uniformed services

Includes members of the police force and defense forces.

#### Long distance drivers

Includes long distance truck drivers and intercity bus drivers.

## Results

### Target groups and background variables

Table 2 shows the characteristics of the study sample (N = 14,524). Of the total sample, 35.8% were selected from population in ANC catchment areas and 11.17% were road construction workers, 11.2% were pastoralists, 9.6% were female sex workers, 9% were police, and 7.4% were members of the defense force. The remaining groups accounted for less than 5% each. The majority of the people in the sample were males (63.6%). Some groups did not have females in the sample (Long distance drivers and to some extent the uniformed services and road construction workers).

**Table 2:** Characteristics of the study populations, behavioral surveys on HIV, Ethiopia, 2005 (N = 14,524)

**Target groups**	**Number**	**Percent**
Teachers	696	4.79

Population of Ante natal care catchments areas	5,207	35.85

Long distance truck drivers	600	4.13

Intercity bus drivers	600	4.13

Pastoralists	1,608	11.07

Road construction workers	1,623	11.17

Female sex workers	1,398	9.63

Police force	1,071	7.37

Defense forces	1,305	8.99

Factory workers	416	2.86

**Age of respondents**		

15 – 24	5,383	38.03

25–34	5,272	37.25

35–59	3,498	24.72

**Education**		

Non literate	4,257	30.28

Primary education	4,521	32.16

Secondary and above	5,279	37.55

**Sex of respondents**		

Female	5,276	36.33

Male	9,246	63.67

**Programmatically important preventive methods (a)**		

Knows none	1,469	10.11

Knows one	1,312	9.03

Knows two	3,440	23.68

Knows three	8,303	57.17

**Misconceptions to common three (b)**		

Have at least one misconception	10,925	75.22

No misconception	3,599	24.78

**misconceptions to six questions**		

No misconception	3,013	20.74

Have misconception_one	2,924	20.13

Have misconcpetion_more than one	8,587	59.12

**Comprehensive knowledge (a+b)**		

No comprehensive knowledge	11,837	81.5

Has comprehensive knowledge	2,687	18.5

**Condom use last sex with paid/paying partner (n = 2,103)**		

Did not use condom	67	3.19

Used condom	2,036	96.81

**Consistent condom use with paid partner (n = 2103)**		

Did not use condom	210	9.99

Used condom	1,893	90.01

**Condom use with non regular partner (n = 668)**		

Did not use condom	162	24.25

Used condom	506	75.75

**Consistent condom use with non regular partner (n = 668)**		

Did not use condom	221	33.08

Used condom	447	66.92

Knowledge of the three preventive methods was 57% of all target groups. Level of misconceptions was measured using two indicators i.e. rejecting three common misconceptions and rejecting six misconceptions. The proportion of people who had at least one misconception out of the three commonly existing misconceptions was very high (75%). Moreover, only one out of five people rejected all six misconceptions. Overall, the proportion of people with comprehensive knowledge was 18.5% (8.9% in females and 24% in males).

### Knowledge and misconceptions

Table 3 indicates distribution of knowledge indicators by sex and target groups. The outcomes included knowledge of prevention methods, level of misconceptions, and level of comprehensive knowledge.

**Table 3:** Knowledge indicators by sex and target group, Ethiopia 2005

**Target group by sex**	**Total**	**Knowledge indicators n (%)**					
		
		**Knowledge of prevention**		**Misconception 3**		**Comprehensive knowledge**	
		**Yes**	**No**	**Yes**	**No**	**Yes**	**No**

**Teachers (696)**							

female	104 (14.9)	59(56.7)	45 (43.3)	60(57.7)	44(42.3)	28(42.3)	76(73.1)

male	592 (85.1)	385(65.0)	207(35.0)	270(45.6)	322(54.4)	233(39.4)	359(60.6)

**ANC population (5207)**							

female	2621 (50.3)	802(30.6)	1819(69.4)	2323(88.6)	298(11.4)	156(6.0)	2465(94.0)

male	2586 (49.7)	1425(55.1)	1161(44.9)	1917(74.1)	669(25.9)	496(19.2)	2090(80.8)

**Long distance truck drivers**							

male	600	457(76.2))	143 (23.8)	394(65.7)	206(34.3)	174(29.0)	426(71.0)

**Intercity bus drivers (600)**							

male	600	469(78.2)	131(21.8)	424(70.7)	176(29.3)	139(23.2)	461(76.8)

**Pastoralists (1608)**							

female	794 (49.4)	147(18.5)	647(81.5)	763(96.1)	31(3.9)	17(2.1)	777(97.9)

male	814 (50.6)	320(39.3)	494(60.7)	722(88.7)	92(11.3)	59(7.2)	755(92.8)

**Road construction workers (1623)**							

female	24 (1.5)	17(70.8)	7(29.2)	14(58.3)	10(41.7)	6(25.0)	18(75.0)

male	1599 (98.5)	1084(67.8)	515(32.2)	1097(68.6)	502(31.4)	383(24.0)	1216(76.0)

**Female sex workers (1396)**							

female	1,396	1060(75.9)	336(24.1)	1171(83.9)	225(16.1)	179(12.8)	1217(87.2)

**Police force (1071)**							

female	162 (15.1)	126(77.8)	36(22.2)	108(66.7)	54(33.3)	45(27.8)	117(72.2)

male	909 (84.9)	613(67.4)	296(32.6)	530(58.3)	379(41.7)	295(32.5)	614(67.5)

**Defense forces (1305)**							

female	17 (1.3)	11(64.7)	6(35.3)	6(35.3)	11(64.7)	9(52.9)	8(47.1)

male	1288 (98.7)	1030(80.0)	258(20.0)	860(66.8)	428(33.2)	331(25.7)	957(74.3)

**factory workers (416)**							

female	158 (38.0)	90(57.0)	68(43.0)	122(77.2)	36(22.8)	28(17.7)	130(82.3)

male	258 (62.0)	208(80.6)	50(19.4)	142(55.0)	116(45.0)	109(42.2)	149(57.8)

**Total (14522)**							

female	5276 (36.3)	2312(43.8)	2964(56.2)	4567(85.6)	709(13.4)	468(8.9)	4808(91.1)

male	9246 (63.7)	5991(64.8)	3255(35.2)	6356(68.7)	2890(31.3)	2219(24.0)	7027(76.0)

Knowledge of the preventive methods ranged between 39.3% in pastoralists to 80.0% in the members of defense forces in males. Among females, pastoralists ranked the lowest for knowledge of preventive methods (18.5%) and female members of the police force ranked the highest (77.8%). Knowledge of preventive methods was consistently low for both females (18.5%) and males (39.3%) in pastoralist communities. Generally, 43.8% of females and 64.8% of males were knowledgeable about preventative methods.

Female pastoralists and female members of the defense forces ranked the highest and lowest (96.1 and 35.3%, respectively) with regards to misconceptions about HIV transmission (though the number of females in the defense forces was very low). Female teachers had a lower level of misconceptions compared to the other groups excluding female members of the defense forces where the number was too small. In general, misconceptions were high both in females and males, 85.6% and 68.7% respectively.

Overall, comprehensive knowledge was generally low (18.5%), 8.9% and 24% for females and males respectively. Amongst women, the level of comprehensive knowledge was lowest among women pastoralists 2.1% and highest among females of the defense forces (52.9%).

### Condom use with non regular partner

Of the total sample, 85.3% (85.8% males and 84.2% females) reported sexual intercourse in the past 12 months prior to the study (not shown in table). Table 4 depicts consistent condom use and condom use during last sexual encounter with non-regular sexual partners and with paying sexual partners among the sexually active. Consistent condom use with non-regular sexual partners was 60.8% for females and 68% for males. Among females, consistent condom use with non-regular sexual partners was lowest among pastoralists (none out of five pastoralists) and was highest among female sex workers (70%). In males, the highest proportion of consistent condom users was among intercity bus drivers (83.7%) and the lowest was among pastoralists (21.4%).

**Table 4:** Condom use indicators by sex and target group, Ethiopia 2005

**Target group by sex**	**Condom use with non regular partner (n = 668)** **n (%)**				**Condom use with paid partner (n = 2103)** **n (%)**			
	
	**Consistent**		**Last sex**		**Consistent**		**Last sex**	
	
	**Yes**	**No**	**Yes**	**No**	**Yes**	**No**	**Yes**	**No**
**Teachers**								

female	3(60.0)	2(40)	3(50.0)	3(50.0)	0	0	0	0

male	31(73.8)	11(26.2)	37(88.1)	5(11.9)	18(85.7)	3(14.3)	19(90.5)	2(9.5)

**ANC population**								

female	12(57.2)	11(47.8)	12(57.2)	11(47.8)	12(80.0)	3(20.0)	14(93.3)	1(6.7)

male	42(56.3)	34(44.7)	51(67.1)	25(32.9)	22(78.6)	6(21.4)	23(82.1)	5(17.9)

**Long distance truck drivers**								

male	31(70.5)	13(29.5)	33(75.0)	11(25.0)	50(80.6)	12(19.4)	60(96.8)	2(3.2)

**Intercity bus drivers**								

male	72(83.7)	14(16.3)	77(89.5)	9(10.5)	54(91.5)	5(8.5)	59(100)	0

**Pastoralists**								

Female	0	5(100)	0	5(100)	0	5(100)	0	5(100)

male	3(21.4)	11(78.6)	5(35.7)	9(64.3)	0	5(100)	0	5(100)

**Road construction workers**								

Female	0	0	0	0	0	0	0	0

male	100(69.0)	45(31.0)	112(77.2)	33(22.8)	84(74.3)	29(25.7)	103(91.2)	10(8.8)

**Female sex workers**								

Female	42(70.0)	18(30.0)	47(78.3)	13(21.7)	1088(93.8)	72(6.2)	1139(98.2)	21(1.8)

**Police force**								

Female	0	0	0	0	0	0	0	0

male	34(63.0)	20(37.0)	42(77.8)	12(22.2)	24(77.4)	7(22.6)	31(100)	0

**Defense forces**								

Female	0	0	0	0	0	0	0	0

male	70(71.4)	28(28.6)	78(79.6)	20(20.4)	536(90.4)	57(9.6)	583(98.3)	10(1.7)

**Factory workers(416)**								

Female	2(50.0)	2(50.0)	2(40.0)	3(60.0)	0	3(100)	0	3(100)

male	5(41.7)	7(58.3)	7(58.3)	5(41.7)	5(62.5)	3(37.5)	5(62.5)	3(37.5)

**Total**								

Female	59(60.8)	38(39.2)	64(66.0)	33(34.0)	1100(93.0)	83(7.0)	1153(97.5)	30(2.5)

male	388(68.0)	183(32.0)	422(76.6)	129(23.4)	793(86.2)	127(13.8)	883(96.0)	37(4.0)

NB. Condom use calculated among sexually active in the past 12 months

Condom use during last sexual encounter with non-regular sexual partners was 66% for females and 76.6% for males. Condom use at last sexual encounter was lowest among female pastoralists (none reported use but numbers were small) and the highest was amongst female sex workers (78.3%). In males, condom use at last sexual encounter was lowest in male pastoralists and highest among intercity bus drivers (89.5%).

### Condom use with paid partner

Among males, consistent condom use was highest (90.4%) in members of the defense forces and lowest among factory workers (62.5%). In general, consistent condom use with paying sexual partners was high (93.0% for females and 86.2% for males). This was skewed towards females because of the female sex workers.

Condom use at last sex with a paying partner was 97.5% for females and 96% for males. Condom use during last sexual encounter was lowest for female pastoralists and factory workers (numbers were too small) while the highest was for female sex workers. Among males, condom use during last sexual encounter was highest among members of the defense force (98.3%) and lowest for factory workers (62.5%).

### Analysis of knowledge vs. target groups

Table 5 indicates the socio-demographic variables by composite indicators of knowledge with adjusted odds ratios. Long-distance truck drivers (OR = 2.07 95% CI = 1.68, 2.55), intercity bus drivers (OR = 2.17 95% CI 1.76, 2.69), road construction workers (OR = 1.42 95% CI 1.23, 1.64), female sex workers (OR = 7.69 95% CI = 6.57, 9.01), members of the police force (OR = 1.40 95% CI = 1.19, 1.64), members of the defense forces (OR = 2.57 95% CI = 2.19, 3.02) and factory workers (OR = 2.36 95% CI = 1.84, 3.02) were more likely than people in ANC catchment areas to mention the three preventive methods. In fact, female sex workers were seven times more likely than people who live in ANC catchment areas to mention the three preventive methods. And members of the defense forces, long-distance drivers, intercity bus drivers and factory workers were at least two times more likely than people in the ANC catchment areas to mention the three preventive methods.

**Table 5:** Socio-demographic variables by three composite indicators of knowledge factors(n = 13803), Ethiopia 2005

**Socio-demographic variables**	**Has knowledge of prevention** **n (%)**				**Rejected 3 common misconceptions** **n (%)**				**Has comprehensive Knowledge** **n (%)**			
	
	**AOR**	**[95% CI]**		**P**	**AOR**	**[95% CI]**		**P**	**AOR**	**[95% CI]**		**P**
***Target group*** teachers	0.96	0.80	1.16	0.68	1.93	1.61	2.31	0.00	1.64	1.36	1.99	0.00

long distance truck drivers	2.07	1.68	2.55	0.00	1.19	0.98	1.46	0.08	1.45	1.18	1.79	0.00

Intercity bus drivers	2.17	1.76	2.69	0.00	0.88	0.72	1.08	0.22	0.99	0.79	1.23	0.90

Pastoralists	1.06	0.92	1.22	0.46	0.81	0.65	1.00	0.05	0.86	0.65	1.12	0.26

road construction workers	1.42	1.23	1.64	0.00	1.13	0.98	1.31	0.10	1.17	1.00	1.37	0.05

female sex workers	7.69	6.57	9.01	0.00	1.52	1.26	1.83	0.00	2.11	1.70	2.61	0.00

police force	1.40	1.19	1.64	0.00	1.39	1.19	1.62	0.00	1.48	1.25	1.75	0.00

defense forces	2.57	2.19	3.02	0.00	1.22	1.05	1.41	0.01	1.28	1.08	1.50	0.00

factory workers	2.36	1.84	3.02	0.00	1.63	1.29	2.05	0.00	2.27	1.78	2.88	0.00

ANC catchment area population*	1.00				1.00				1.00			

***Education***												

non literate	0.24	0.22	0.27	0.00	0.20	0.17	0.23	0.00	0.17	0.14	0.20	0.00

primary education	0.66	0.60	0.72	0.00	0.46	0.42	0.51	0.00	0.47	0.42	0.52	0.00

high school and higher*	1.00				1.00				1.00			

***age group***												

15–24*	1.00				1.00				1.00			

25–34	1.00	0.92	1.10	0.92	0.94	0.86	1.04	0.24	0.90	0.81	1.00	0.06

35–59	0.74	0.67	0.82	0.00	0.77	0.69	0.87	0.00	0.70	0.62	0.80	0.00

***sex ***												

Female*	1.00				1.00				1.00			

male	1.99	1.81	2.20	0.00	1.98	1.75	2.24	0.00	2.37	2.04	2.74	0.00

AOR = adjusted odds ratio; * = referent group

Correct identification of the three questions on misconceptions about HIV was statistically significantly higher for teachers (OR = 1.93, 95% CI = 1.61, 2.31), female sex workers (OR = 1.52, 95% CI 1.26, 1.83), members of the police forces (OR = 1.39 95% CI 1.19, 1.62) members of the defense forces (OR = 1.22 95% CI = 1.05, 1.41), and factory workers (OR = 1.63 95% CI = 1.29, 2.05). Correct identification of misconceptions was marginally significantly lower amongst pastoralists than people who live in ANC catchment areas (OR = 0.61 95% CI = 0.65, 1.00).

Teachers (OR = 1.64 95% CI = 1.6, 1.99), long distance truck drivers (OR = 1.45 95% CI = 1.18, 1.79), road construction workers (OR = 1.17 95% CI = 1.00, 1.37), female sex workers (OR = 2.11 95% CI = 1.10, 2.61), members of the police force (OR = 1.48 95% CI = 1.25, 1.75), members of the defense forces (OR = 1.28 95% CI = 1.08, 1.50), and factory workers (OR = 2.27 95% CI = 1.78, 2.88) were more likely than the people who live in ANC catchment areas to have comprehensive knowledge. Female sex workers and factory workers were two times more likely than the people in ANC catchment areas to have comprehensive knowledge.

With regards to socio-demographic factors, the non-literate and those with primary education were less likely than those with high school and above education to have knowledge of preventive methods (OR = 0.24 95% CI = 0.22, 0.27; OR = 0.66 95% CI = 0.60, 0.72), reject common misconceptions (OR = 0.20 95% CI = 0.17, 0.23; OR = 0.46 95% CI = 0.42, 0.51), and have comprehensive knowledge about HIV & AIDS (OR = 0.17 95% CI = 0.14, 0.20; OR = 0.47 95% CI = 0.42, 0.52). Males were more likely than females to have better knowledge on preventive methods (OR = 1.99 95% CI = 1.81, 2.20), reject common misconceptions (OR = 1.98 95% CI = 1.75, 2.24), and have comprehensive knowledge of HIV & AIDS (OR = 2.37 95% CI = 2.04, 2.74).

People 35 years and older were less likely than those 15 – 24 years of age to have knowledge of preventive methods (OR = 0.74 95% CI = 0.67, 0.82), reject common misconceptions (OR = 0.77 95% CI = 0.69, 0.87), and have comprehensive knowledge (OR = 0.70 95% CI = 0.62, 0.80).

### Analysis of condom use by target group

Table 6 shows condom use with non regular and paying sexual partners against the different target groups. In our analysis, age, sex and education were controlled while the people who live in ANC catchment areas was used as the referent group.

**Table 6:** Consistent condom use and condom use at last sex with non regular partner 12 months and paid sex partner by target group, Ethiopia 2005

***Target group***	**Non regular partner (n = 668)**								**Paid partner (n = 2103)**							
	
	**Consistent condom use**				**Condom use at last sex**				**Consistent condom use**				**Condom use at last sex**			
	
	**AOR**	**[95% CI]**		**P**	**AOR**	**[95% CI]**		**P**	**AOR**	**[95% CI]**		**P**	**AOR**	**[95% CI]**		**P**
Teachers	1.61	0.74	3.51	0.23	2.08	0.81	5.30	0.13	1.40	0.30	6.51	0.67	1.52	0.25	9.40	0.65

long distance truck drivers	2.03	0.89	4.63	0.09	1.57	0.65	3.77	0.31	1.08	0.35	3.33	0.89	5.96	1.05	33.80	0.04

Intercity bus drivers	4.15	1.98	8.69	0.00	4.06	1.73	9.53	0.00	2.59	0.71	9.45	0.15				

Pastoralists	0.44	0.11	1.81	0.25	0.52	0.14	1.85	0.31								

road construction workers	2.11	1.16	3.85	0.02	1.84	0.96	3.56	0.07	0.73	0.26	2.02	0.54	2.45	0.69	8.67	0.16

female sex workers	3.11	1.19	8.14	0.02	5.21	1.89	14.33	0.00	4.56	1.40	14.84	0.01	5.98	1.15	31.05	0.03

police force	1.07	0.52	2.20	0.86	1.26	0.56	2.85	0.58	0.80	0.23	2.80	0.73				

defense forces	2.14	1.12	4.10	0.02	1.98	0.97	4.03	0.06	2.28	0.87	5.97	0.09	11.91	3.56	39.85	0.00

factory workers	0.51	0.17	1.50	0.22	0.55	0.18	1.66	0.29	0.30	0.06	1.46	0.14	0.22	0.04	1.15	0.07

AOR = adjusted odds ratio for sex, education and age; Referent group = population of ANC catchment areas; NB. Condom use calculated among sexually active in the past 12 months

Consistent condom use with non-regular sex partners was significantly higher among intercity bus drivers (OR = 4.15 95% CI = 1.98, 8.69), road construction workers (OR = 2.11 95% CI = 1.16, 3.85), female sex workers (OR = 3.11 95% CI = 1.19, 8.14) and members of the defense forces (OR = 2.14 95% CI = 1.12, 4.10) than it was among people in ANC catchment areas. Intercity bus drivers (OR = 4.06 95% CI = 1.73, 9.53) and female sex workers (OR = 5.21 95% CI = 1.89, 14.33) were more likely than people in the ANC catchment areas to report condom use during last sexual encounter with non-regular partners. For members of the defense forces the likelihood of condom use at last sex was marginally significant (p = 0.06).

Female sex workers were more likely than people in the ANC catchment area to report consistent condom use with paying partners (OR = 4.56 95% CI = 1.40, 14.84). Consistent condom use was not statistically significantly different for the rest of the study groups compared to the people who live in ANC catchment areas. On the other hand, long-distance truck drivers (OR = 5.96 95% CI = 1.05, 33.80), female sex workers (OR = 5.98 95% CI = 1.15, 31.05) and members of the defense force (OR = 11.91 95% CI = 3.56, 39.85) were more likely than people in ANC catchment areas to report condom use during last sexual encounter with paying partners. Female sex workers were five times more likely than the people in ANC catchment areas to report consistent condom use with paying sexual partners.

## Discussion

This analysis provides a better understanding of the level of knowledge of HIV among important sub-populations in Ethiopia that are considered to be primary HIV intervention targets for the Ethiopian government.

### Knowledge of preventive methods

Generally, knowledge of the preventive methods was fairly high; however, considerable knowledge gaps were observed among rural, females, people 35 years and older and the illiterate segments of the studied population. The Ethiopian DHS conducted in 2005 revealed that knowledge of preventive methods for limiting sex to one uninfected partner and use of condoms was 65.5% for urban women and 28.0% for rural women, and was 75.5% for urban men and 53.3% for rural men [4]. In Jimma town and its neighboring communities, nearly two-thirds of the respondents earned an adequate knowledge score [15]. Although the figures were not the same due to the difference in the nature of the studies, the pattern in this study was similar – women and groups with less access to information were less knowledgeable on ways to prevent HIV. Knowledge of preventive methods exceeded 66% among groups that are generally stigmatized for maintaining the HIV epidemic in Ethiopia such as long distance drivers, female sex workers, and uniformed services that expressed established sexual networks. Groups such as construction workers also demonstrated considerable knowledge which may be due to policy influences as a result of mainstreaming of HIV in sectors and labor laws that demand HIV intervention activities in all development sectors in Ethiopia [16,17].

Further, our analysis indicates that knowledge of preventive methods was significantly higher in groups that had better access to information and interventions; however it was lower among pastoralists who had limited access to modern media such as printed material, radio and television. This demonstrates the need to design alternative strategies to improve access to correct information to communities such as the pastoralists who are largely mobile. Programs should also implement tailored strategies to address the needs of the non-literate and women. Our study revealed that knowledge of preventive methods was lower among people 35 years and older compared to people 15–24 years of age. From the different studies conducted on adolescents and youth, knowledge was considered as adequate in terms of knowing the three programmatically important preventive methods [18-20].

### Misconceptions

The magnitude of misconceptions was high in almost all groups but very high in groups that did not score well in levels of knowledge of preventive methods. Other authors have reported that there existed considerable levels of misconceptions about the transmission of HIV and prevention methods [18]. Other segments of the population such as people in the informal business sector in Addis Ababa have considerable misconceptions about the transmission of HIV [19]. In Northwest Ethiopia, it was reported that although knowledge on HIV was reasonably high, considerable misconceptions were noted among the study population [20]. To this end, it was also surprising to see high levels of misconceptions among female sex workers, as their knowledge of preventive methods was relatively high. It is important to note that female sex workers and long distance truck drivers were the first groups put under biological as well as behavioral surveillance in Ethiopia [21,22]. However, it is likely that the prevention programs focused on prevention methods and paid little attention to address misconceptions.

The proportion of female sex workers that had comprehensive knowledge was very low. This was attributed to the presence of widespread misconceptions about the transmission of HIV infection [13,14]. Nevertheless, the major difficulty in comparing knowledge across studies is the variability in definitions as well as the breadth of measurement regarding HIV knowledge. The scope of measurement extends from assessing simple awareness to the development of an index measuring the different domains of knowledge about HIV & AIDS. Behavioral surveillance surveys define comprehensive knowledge by combining knowledge of preventive methods with the ability to reject prevailing common misconceptions. In this regard, authors have also indicated the importance of addressing incorrect information in promoting condoms and building self-efficacy [23,24].

### Condom use

Condom use in every penetrative sexual act has remained one of the major indicators of behavior change in HIV/AIDS preventions. In this study condom use was measured using two indicators: 1) report of condom use during the last sexual encounter with non-regular sexual partners or paying sexual partners; and 2) pattern of condom use in any sexual relationship in the last 12 months (does not include condom use among married couples), which was described as consistent condom use.

The findings on female sex workers in terms of reporting condom use during last sexual encounter with a paying partner and consistent condom use with a non-regular sexual partner was consistently high. This might indicate changes in behavior among high-risk groups such as female sex workers. Consistent condom use and condom use during last sexual encounter with paying sexual partners did not show statistically significant difference in all the groups except female sex workers. This might mean that the pattern of condom use with high-risk partners such as female sex workers was similar across all study groups. In a randomized controlled trial in high-risk settings in Nicaragua, it was demonstrated that condom use was significantly higher in paid sex than non-commercial sex [25]. Similarly, a higher proportion of female sex workers reported consistent condom use with paying clients (77%) in Assossa, Ethiopia [26]. However, these studies are at least three to four years old compared to the findings of our study. On the other hand, other studies have shown that consistent condom use was low among brothel-based female sex workers in India and Ethiopia [27-29].

In our study condom use among long truck drivers was high compared to other reports. Among 343 long distance truck drivers in Bangladesh less than a third (31%) had ever used a condom which was very low [30]. Among female sex workers, the practice of condom use varied by type of partner with a tendency of low utilization within sexual relationships to long-term partners (as compared to casual partners). This was also reported by earlier studies [31]. Others have indicated that condom use with casual partners was determined by the interpersonal interaction between partners and less by attitudes and beliefs (unlike with established partners) [32]. The negotiations whether to request a partner to use or not to use condoms relies on several factors including the type of partner, access to condoms, a priori knowledge and risk perception. Long term sexual relationships tend to build norms and understanding among partners. In the behavioral surveillance surveys one of the major reasons for not using condoms was trusting a partner [13].

## Conclusion

Knowledge of preventive methods was fairly high in almost all the target groups. However, there exists high level of misconception about HIV/AIDS among the target groups selected in this study. The misconceptions were greatest among groups with less access to media such as pastoralists which indicates a need to devise special strategies to address misconceptions among those without access to conventional forms of media.

The level of comprehensive knowledge was very low across all target groups but severe among pastoralists, women, people above the age of 35, as well as those who cannot read and who lives in rural areas. These deficiencies also highlight the limited efforts taking place to address in misconceptions and teach these sub-populations about prevention methods.

Consistent condom use and condom use during last sexual encounter was consistently high among female sex workers both with paying sexual partners as well as with non-regular sexual partners. Members of the defense force show a similar pattern indicating possible positive outcome of the HIV/AIDS interventions. Consistent condom use with paying partners was not statistically significant among the different target groups with the exception of female sex workers. This also might be a positive sign, though considerable proportions did not use condoms with non-regular partners. The HIV/AIDS prevention program must continue to encourage people to use condoms during sexual relations with different types of sexual partners.

## Competing interests

The authors declare that they have no competing interests.

## Authors' contributions

GM is principal investigator of BSS in Ethiopia, developed the protocol, conducted the analysis of the secondary data and did the write up of the manuscript; DH contributed in the development of the protocol, analysis of the data and reviewed the manuscript; AM contributed in the conceptual design of the analysis of the data and in the writing of the manuscript.

## Pre-publication history

The pre-publication history for this paper can be accessed here:


